# Corrosion behavior of X80 pipeline steel local defect pits under static liquid film

**DOI:** 10.1038/s41598-021-99973-8

**Published:** 2021-10-21

**Authors:** Zhuowei Tan, Zhenbo Wang, Shengzhu Zhang, Shuyu Bai, Dalei Zhang, Youhai Jin, Shaohua Xing

**Affiliations:** 1 China University of Petroleum (East China), Shandong, 266580 China; 2 Xiangtan University, Hunan, 411105 China; 3 China Academy of Safety Science and Technology, Beijing, 100012 China; 4 School of Materials Science and Engineering, China University of Petroleum (East China), Shandong, 266580 China; 5 State Key for Marine Corrosion and Protection, Luoyang Ship Material Research Institute, Qingdao, 266237 China

**Keywords:** Fluidics, Metals and alloys

## Abstract

In this work, the corrosion electrochemical information under different thicknesses of liquid film was tested. The local corrosion development process of X80 steel under different thicknesses of liquid film was studied by combining the detection and analysis of scale and the matrix corrosion morphology. The corrosion was studied by EIS. The composition and microstructures of corrosion scale at different locations were detected by EDS and SEM, and the metal matrix was detected by 3D topography technology to analyze the local corrosion. The results show that a liquid film with a thickness greater than or equal to 1 mm has no effect on the mechanism of the corrosion process, but has a control effect on the corrosion rate and the time of each stage in corrosion. The corrosion process can be divided into two stages: in the early stage, the concentration of ions inside and outside ADP is the same, so the corrosion is uniform; in the later stage, due to the influence of CO_2_ dissolution and mass transfer distance, the cathodic reaction is mainly outside ADP and the anodic reaction is mainly inside ADP. In addition, corrosion acidification occurs in ADP, which enhances the corrosion process in ADP.

## Introduction

The CO_2_ corrosion of natural gas transportation pipelines poses a significant threat to the operation of the pipeline^1–4^. A pure gas environment usually does not cause corrosion of pipeline steel. However, natural gas collection usually contains a certain amount of water vapor, which condenses to form a liquid film during transportation, forming a corrosive environment^5–8^. Therefore, corrosion under the liquid film is the main form of corrosion in natural gas transportation pipelines.

There has been a lot of research on the corrosion process under the liquid film of pipeline steel. Many researchers focus on the influence of the flow field under the dynamic liquid film on the corrosion mass transfer process and the integrity of the corrosion product film. The research results show that the dynamic liquid film can effectively promote the corrosion mass transfer process. However, the microstructure and composition of the corrosion product film are also affected by the mechanical effect of the flow field. Therefore, the acceleration effect of the flow field on the corrosion under dynamic film is not a simply positive growth process^9,10^. Otherwise, the influence of liquid film thickness on corrosion has also been studied. Some studies show that when the liquid film thickness reaches a certain range, the CO_2_ corrosion of X80 steel will develop from highly localized pitting corrosion to uniform corrosion^11^. When the liquid film thickness increases from 100 to 1000 μm, the cathodic process of CO_2_ corrosion of X52 steel changes from activation control to mass transfer control, and the corrosion rate is proportional to the liquid film thickness. Further studies show that CO_2_ corrosion under liquid film is affected by CO_2_ solubility and corrosion product film. The corrosion process is mainly controlled by cathodic diffusion, and the thickness of diffusion layer on the electrode surface is less than 1000 μm^12^.

However, the surface morphology defects in the pipeline will lead to differences in ion distribution, mass transfer distance, and electrochemical environment in different regions, which play a decisive role in the development of local corrosion. There are very few studies on this issue. In this work, X80 pipeline steel samples with arc defect pit (ADP) were used to simulate local corrosion pits. The corrosion electrochemical information under different thicknesses of liquid film was tested, and the local corrosion development process of X80 steel under different thicknesses liquid film was studied by detection and analysis of corrosion products and metal matrix corrosion morphology. The results show that a liquid film with a thickness greater than or equal to 1 mm has no effect on the mechanism of the corrosion process, but has a control effect on the corrosion rate and the time of each stage in corrosion. At the same time, the local corrosion under laboratory conditions is the overall enhanced local corrosion in the ADP.

## Experimental

### Setup

**Figure 1:**
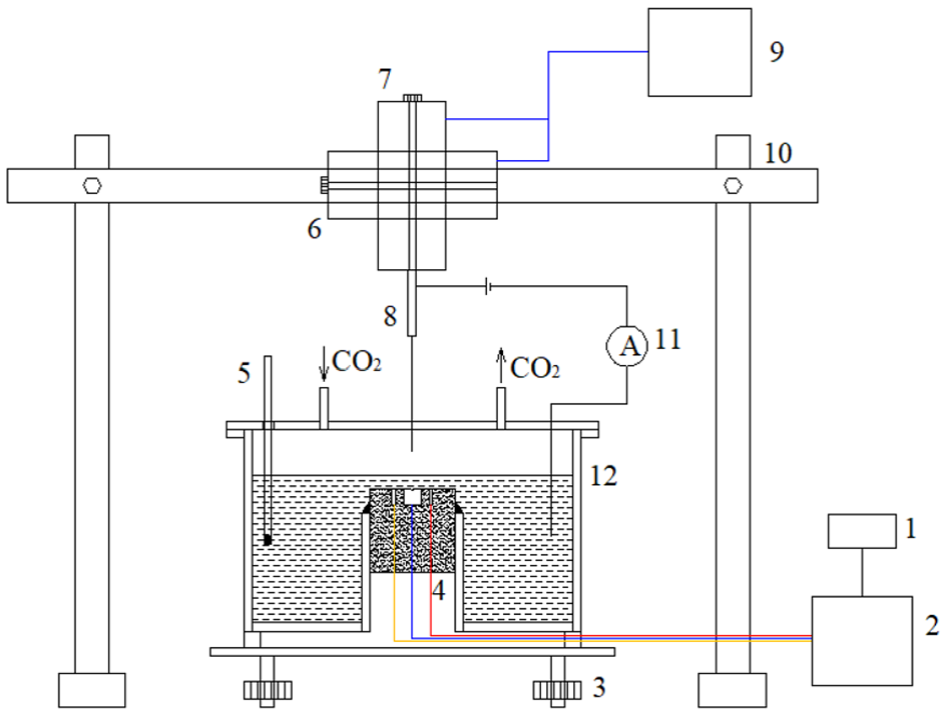
Static Corrosion test platform under liquid film: (1) data acquisition computer, (2) electrochemical workstation, (3) adjustment nut, (4) testing electrode, (5) thermometer, (6) horizontal displacement mechanism, (7) vertical displacement mechanism, (8) conductivity probe, (9) displacement control computer, (10) gantry frame, (11) micro ammeter, (12) solution container.

The static liquid film testing and corrosion electrochemical testing system as shown in Fig. 1. The system mainly consists of the liquid film thickness measurement system, solution container, horizontal adjustment platform and electrochemical workstation. The liquid film thickness measurement system consists of a two-dimensional translation probe and a micro current loop. The solution container is a cylindrical container with an upwardly protruding test sample mounting port at the bottom of the center, which is used to install an integrated electrochemical test probe. The integrated electrochemical test probe is described in the previous work^9,10^. The upper cover of the solution tank is provided with a number of small ports, which are used to set thermometers, micro current loop probe, CO_2_ gas inlet, and CO_2_ gas outlet.

### Materials and preparation

**Figure 2:**
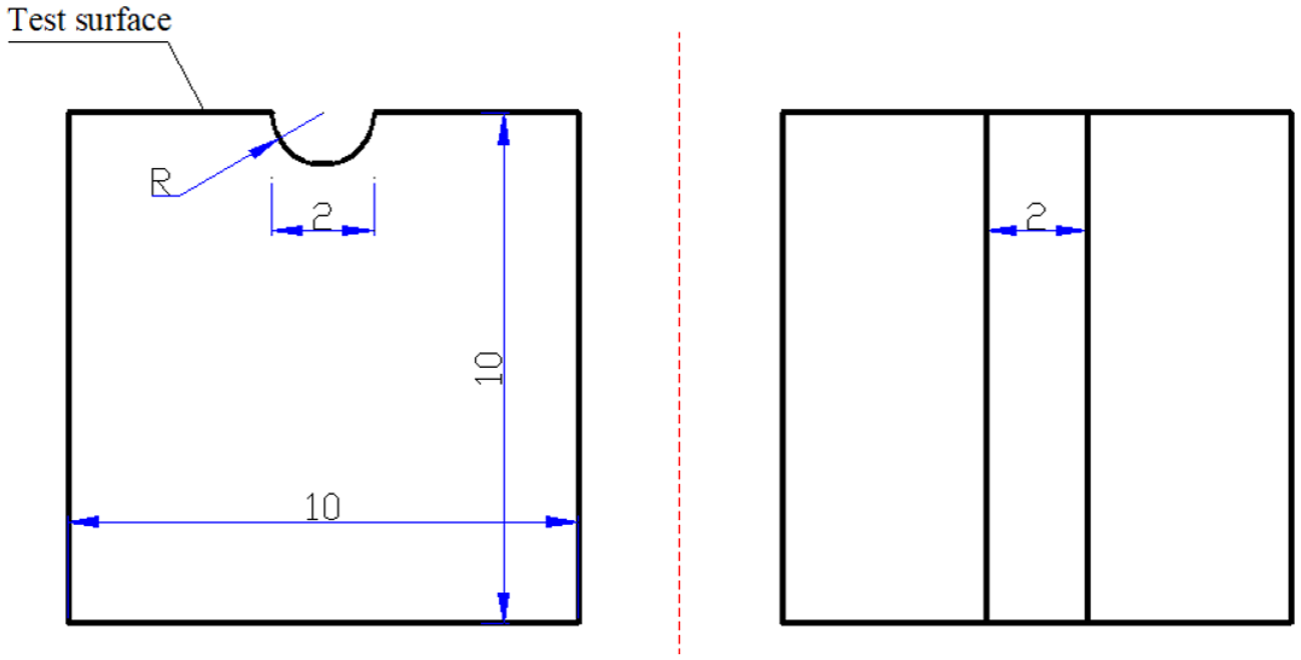
Specification of ADP: left: side view, right: top view, unit: mm.

The X80 pipeline steel (chemical composition listed in Table 1) was processed into cubic corrosion test samples with side length of 1 cm first. ADP with width 2 mm and radians 1 mm were machined on test surfaces of corrosion samples which was used to simulate local corrosion pits (Fig. 2). All samples were sequentially ground with 400, 600, 800 and 1000 grit silicon carbide sand papers followed by degreasing with acetone, and then rinsing with deionized water. All samples for corrosion test in this study were taken from the same piece of X80 pipeline steel, and the low temperature cutting technology was used to avoid the thermal stress or change of metallographic structure. At the same time, take the inner surface of the pipeline steel as the same side of the test surface, so as to ensure that the pipeline steel samples metallographic structure and heat treatment of all test samples are the same as that of the actual operation of the pipeline.

**Table 1:** Chemical element composition of X80 pipeline steel (wt%).

X80 pipeline steel (balance Fe)										
Mn	Si	Mo	C	Nb	V	Cr	Ni	Ti	P	S
1.86	0.28	0.22	0.063	0.061	0.059	0.03	0.03	0.016	0.011	0.0006

In this work, the saturated CO_2_-NACE solution was used as the corrosion test solution, and the solution composition and mass ratio were H_2_O:NaCl:CH_3_COOH = 94.5:5:0.5. After the NACE solution is prepared in the solution tank according to the proportion of components, N_2_ is purged to remove oxygen for 4 h first, and then high-purity CO_2_ gas is purged to make the solution CO_2_ saturation. During the whole test period, keep CO_2_ continuously purged into the upper part of static liquid film to ensure that the solution is always in CO_2_ atmosphere. All corrosion tests were conducted at 40 ± 1 °C under atmospheric pressure.

### Electrochemical measurements

The electrochemical measurements were conducted in a three-electrode system. X80 pipeline steel samples were used as WE, platinum as CE, and high purity zinc as RE. The electrochemical measurements were carried out on a Solartron 1287 + 1255B workstation. The corrosion time of each group was set to 16 h. The open-circuit potential (OCP) of X80 steel WE were monitored before each test to ensure that the system is in a stable state during each test. Electrochemical impedance spectroscopy (EIS) measurements were performed at sinusoidal potential excitation of 5 mV at frequencies varying from 1 MHz to 10 mHz.

### Surface analysis

After the corrosion test, rinse the samples with deionized water and dried in a vacuum drying oven. The test surface is divided into two areas: the outer plane of the defect pit and the inner area of the defect pit, labeled as areas a and b respectively. The corrosion products and corrosion morphology are detected according to the two areas. The micro morphology and element distribution of corrosion products on the surface were obtained by FEI QUANTA-FEG2500 scanning electron microscope (SEM) and energy dispersive X-ray spectroscopy (EDS). The local corrosion of the substrate was measured by laser 3D morphology after removing the corrosion scale.

## Results and discuss

### Electrochemical measurements

**Figure 3:**
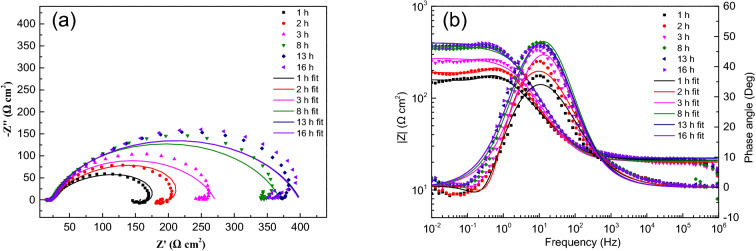
EIS spectra for sample with ADP under static liquid film, h = 1 mm: (**a**) Nyquist plots, (**b**) Bode and |Z| plots.

**Figure 4:**
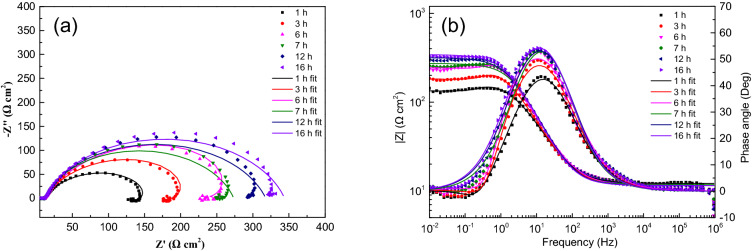
EIS spectra for sample with ADP under static liquid film, h = 2.5 mm: (**a**) Nyquist plots, (**b**) Bode and |Z| plots.

**Figure 5:**
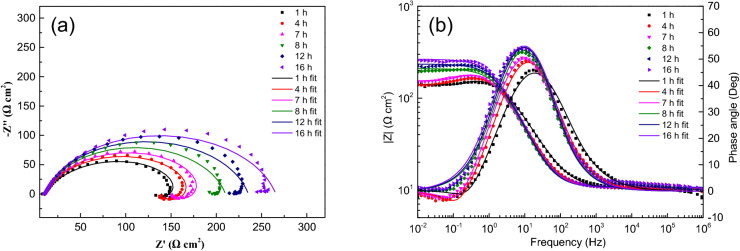
EIS spectra for sample with ADP under static liquid film, h = 4.5 mm: (**a**) Nyquist plots, (**b**) Bode and |Z| plots.

**Figure 6:**
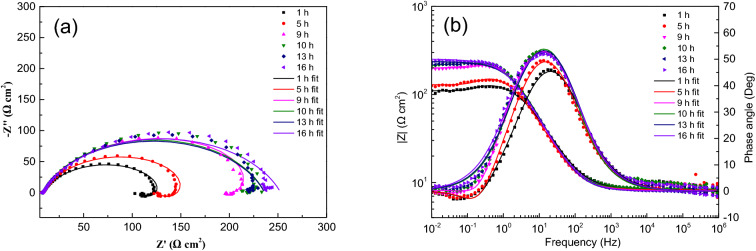
EIS spectra for sample with ADP under static liquid film, h = 6 mm: (**a**) Nyquist plots, (**b**) Bode and |Z| plots.

**Figure 7:**
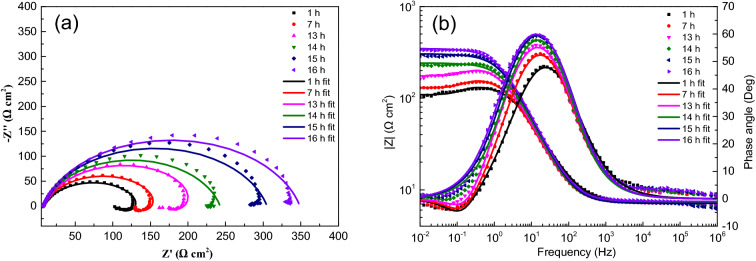
EIS spectra for sample with ADP under static liquid film, h = 8 mm: (**a**) Nyquist plots, (**b**) Bode and |Z| plots.

**Figure 8:**
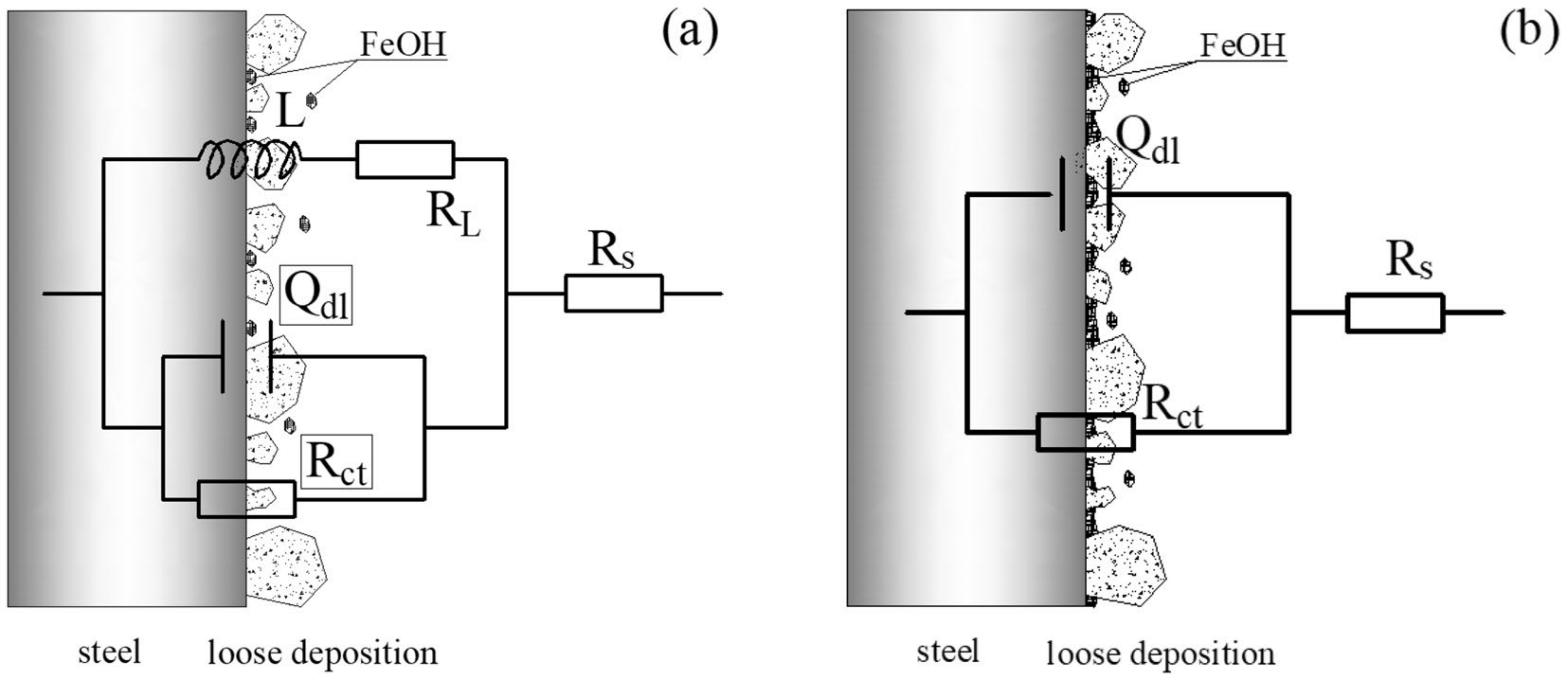
Equivalent circuit of EIS data of samples with ADP under static liquid film: (**a**) Early stage of corrosion, (**b**) Later stage of corrosion.

The test data and fitting curve of EIS obtained with corrosion progress are shown in Figs. 3, 4, 5, 6, 7. All the EIS data under different thickness liquid film show two different EIS characteristics, a high frequency capacitive semicircle and a low frequency inductive loop were identified in the early stage of corrosion, and a single capacitive semicircle shown in the later stage. The interface dispersion effect caused by the roughness of the electrode surface makes the measured capacitive semicircle is not a geometric semicircle^13^. The analysis software Zsimpwin is used to fit the experimental data, and the equivalent circuit diagram is shown in Fig. 8, where R_S_ is the solution resistance, R_CT_ is the charge transfer resistance, L is the inductance, R_L_ is the inductive reactance, and Q_dL_ is the constant phase element (CPE) related to the double layer capacitive, and the impedance can be calculated by formula (1). ^14^.

1 $${Z}_{CPE}=\frac{1}{{(Qj\omega )}^{n}}$$

where Q is a proportional factor, j equals to (-1)^1/2^, ω represents frequency, and n is a factor with values between 0 and 1.

The impedance parameters obtained by fitting are listed in Table 2. The impedance spectra of corrosion reaction with time under different thickness liquid film have similar characteristics, which proves that under the experimental conditions set in this study, the corrosion mechanism of X80 steel under different thickness liquid film are basically the same, and the liquid film thickness only affects the time of each step of corrosion reaction.

**Table 2:** Equivalent circuit fitting data of EIS data of samples with ADP under static liquid film.

Thickness (mm)	Time (h)	R_s_ (Ω cm^2^)	Q_dl_ (Ω^-1^ cm^-2^ s^–n^)	n_dl_	R_ct_ (Ω cm^2^)	L (H cm^-2^)	R_L_ (Ω cm^2^)
1	1	20.92	9.8E−4	0.6614	210.4	234.7	406.3
	2	20.00	8.7E−4	0.6747	298.0	168.3	411.4
	3	20.53	4.5E−4	0.7869	250.3		
	8	20.69	3.6E−4	0.8008	349.5		
	13	21.78	3.9E−4	0.7872	378.3		
	16	22.36	3.9E−4	0.7910	376.2		
2.5	1	12.11	7.7E−4	0.7507	159.4	356.7	527.9
	3	11.59	7.2E−4	0.7580	249.6	259.6	552.7
	6	11.49	6.8E−4	0.7488	365.5	264.8	653.2
	7	11.78	4.6E−4	0.8248	261.7		
	12	11.48	5.0E−4	0.8065	306.3		
	16	11.49	5.0E−4	0.8124	332.4		
4.5	1	10.14	6.9E−4	0.7519	167.6	355.5	582.9
	4	10.34	7.2E−4	0.8064	175.3	636.9	514.5
	7	10.53	7.8E−4	0.8171	189.5	745.2	609.5
	8	10.66	6.6E−4	0.8539	199.5		
	12	10.66	6.3E−4	0.8540	224.7		
	16	10.51	6.3E−4	0.8425	255.2		
6	1	8.35	7.4E−4	0.7689	131.0	442.6	458.8
	5	8.28	7.8E−4	0.789	163.6	394.2	464.7
	9	8.10	7.3E−4	0.789	249.5	660.1	862.3
	10	8.49	6.2E−4	0.8156	227.7		
	13	7.95	6.6E−4	0.8004	228.2		
	16	7.96	7.3E−4	0.7844	244.9		
8	1	7.21	6.6E−4	0.7788	134.9	543.5	417.5
	5	7.10	6.6E−4	0.8152	162.5	581.6	507.8
	9	7.10	6.4E−4	0.8160	220.9	714.0	713.8
	13	7.25	5.2E−4	0.8459	235.5		
	14	7.10	5.0E−4	0.8411	298.0		
	16	7.46	4.9E−4	0.8389	341.4		

### Corrosion scale microstructure and composition

**Figure 9:**
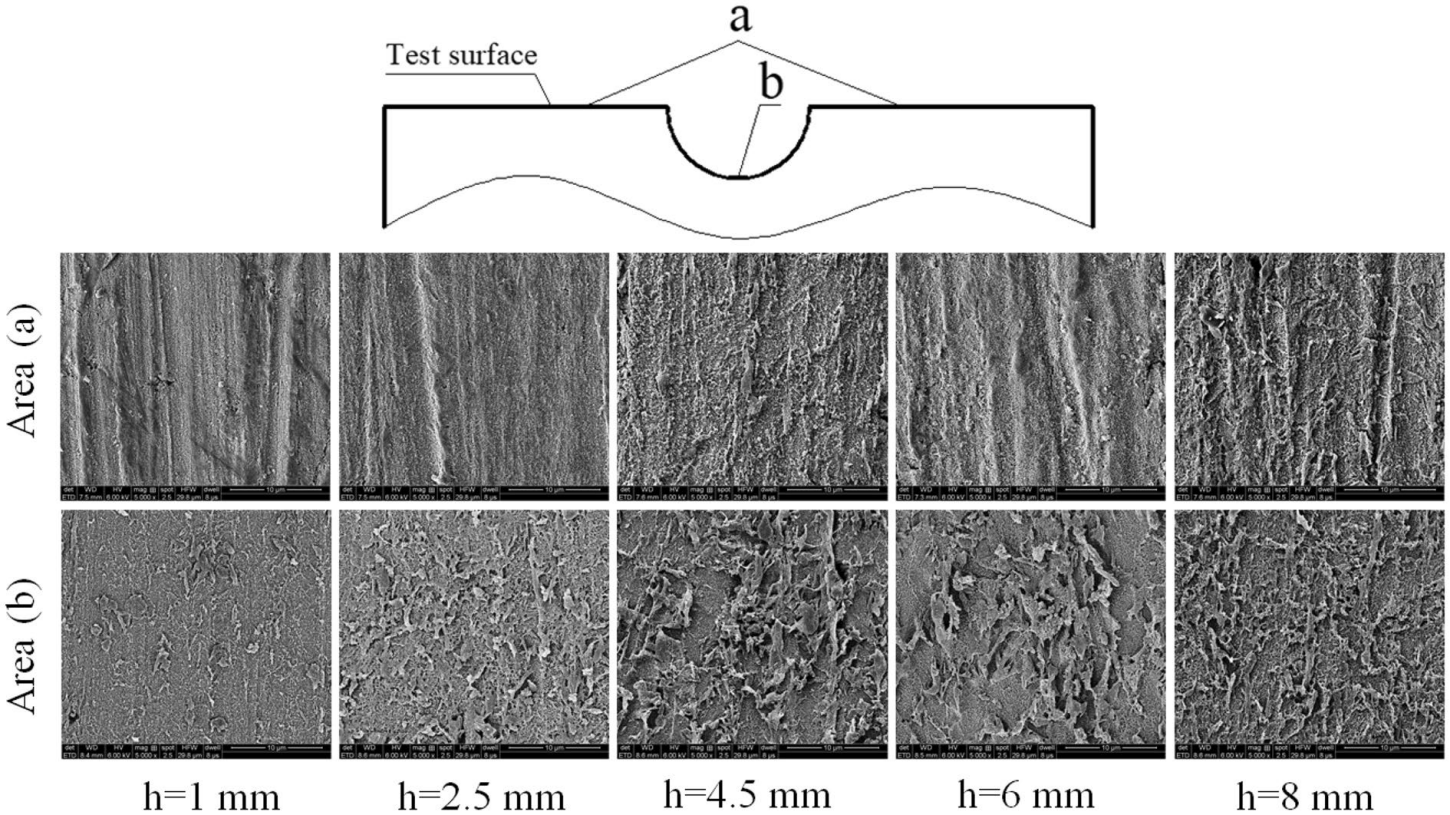
SEM of corrosion products in different area.

The surface morphology of corrosion products on both inside and outside of the ADP after corrosion test is obtained by SEM, and the results are shown in Fig. 9. SEM results show that under different thickness liquid film, corrosion products are formed inside and outside of the ADP. However, the results of different thickness liquid film show that the corrosion products in the ADP are much more than those in the outer plane of the ADP, which proves that the corrosion dissolution process of the matrix is more serious inside of the ADP.

**Figure 10:**
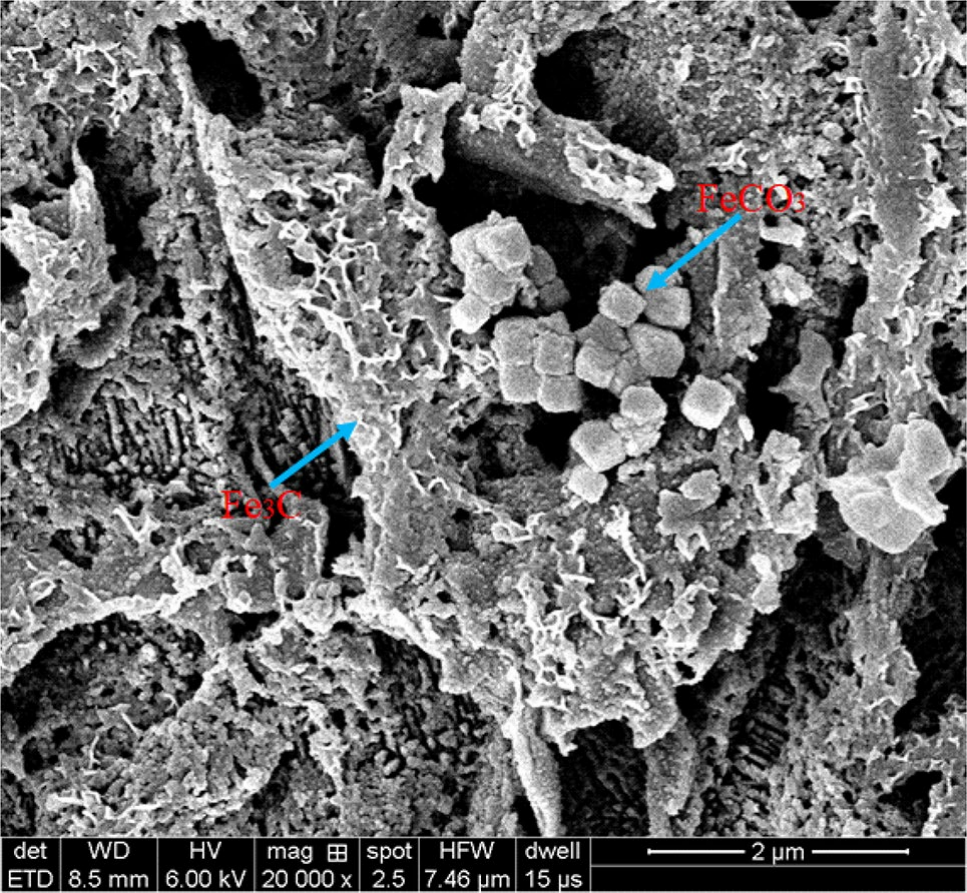
SEM of FeCO_3_ crystal attach to Fe_3_C residue.

The composition of corrosion products is obtained by EDS, and the atomic number ratio of each element obtained are listed in Table 3. The atomic number ratio of elements shows that the atomic ratio of Fe and C is close to 3:1 both inside and outside of the ADP under different thickness liquid film. This is because the ferrite in X80 pipeline steel will be first dissolved by reaction in saturated CO_2_-NACE solution, and the Fe_3_C generated in the smelt process with stable chemical property which will remain on the surface after corrosion. Fe_3_C appear inside and outside the ADP, which indicates that CO_2_ corrosion reaction will occur inside and outside the defect pits when there are local defect pits on the surface in static solution. There are two sources of O element. On the one hand, there is a very small amount of corrosion deposit FeCO_3_ attached to the corrosion residue Fe_3_C, as shown in Fig. 10. On the other hand, after the tested, the sampling and drying process of sample was carried out in the air, which would cause the formation of a few oxides.

**Table 3:** EDS test element content inside and outside defect of defect samples under static liquid film (atom %).

Area	h (mm)	C K	O K	Si K	Mn K	Fe K	Mo k
a	1	25.99	9.55	0.89	1.24	62.14	0.19
	2.5	25.48	10.29	0.72	1.20	62.09	0.22
	4.5	23.02	9.12	1.24	1.46	64.97	0.19
	6	27.43	11.01	1.23	1.29	58.94	0.09
	8	26.34	8.78	1.08	1.21	62.31	0.28
b	1	17.65	12.83	1.07	1.23	66.99	0.22
	2.5	19.31	11.07	0.67	1.30	67.51	0.13
	4.5	20.42	10.68	1.14	1.39	66.23	0.13
	6	21.09	9.57	1.14	1.48	66.51	0.20
	8	25.18	10.94	1.18	1.22	61.30	0.18

### 3D morphology

**Figure 11:**
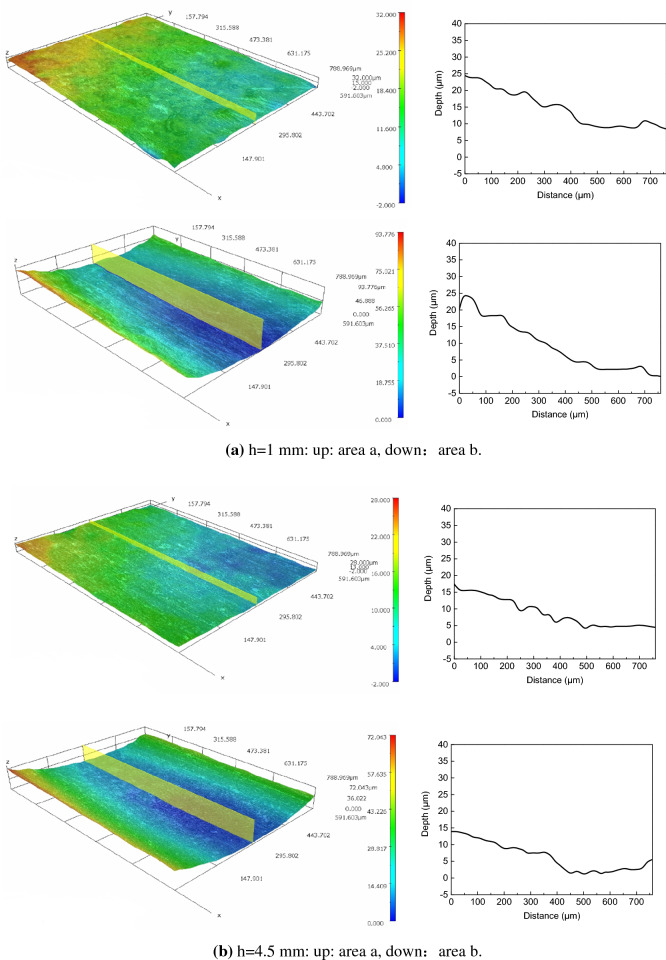

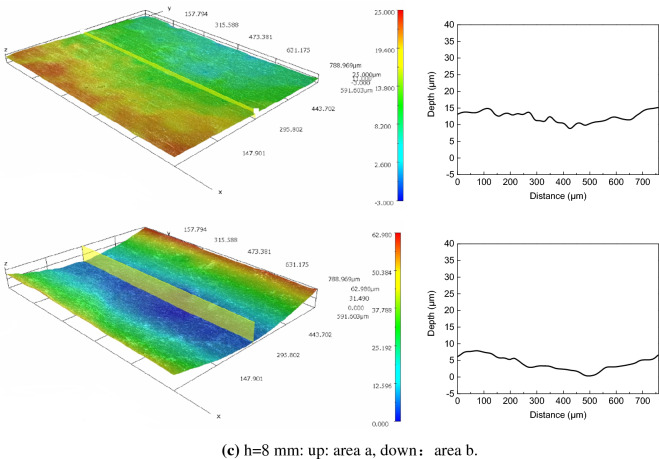
3D surface morphology and profile of the substrate corrosion under static liquid film.

Remove the corrosion products after the corrosion test, and the metal substrate surface is characterized by 3D morphology. The results of 3D morphology and profile characterization are shown in Fig. 11, which show that there are no obvious local corrosion pits inside and outside of the ADP under different liquid film thickness.

## Analysis

### Mechanism of reaction

The corrosion of X80 pipeline steel in saturated CO_2_-NACE solution has a relatively clear corrosion reaction mechanism^15–17^. A large number of studies show that the reaction is greatly affected by temperature and pH value^18^. In this reaction system, the pH value of the solution is 2.7 ± 0.02, the temperature is 40 ± 1 °C, and the cathodic reaction mainly includes the following steps:

2 $$2{\mathrm{H}}_{\mathrm{aq}}^{+}+2{\mathrm{e}}^{-}\to {\mathrm{H}}_{2(\mathrm{g})}$$

3 $$2{\mathrm{H}}_{2}{\mathrm{CO}}_{3(\mathrm{aq})}+2{\mathrm{e}}^{-}\to {\mathrm{H}}_{2(\mathrm{g})}+{2\mathrm{HCO}}_{3(\mathrm{aq})}^{-}$$

4 $${2\mathrm{HCO}}_{3(\mathrm{aq})}^{-}+2{\mathrm{e}}^{-}\to {\mathrm{H}}_{2(\mathrm{g})}+{2\mathrm{CO}}_{2(\mathrm{aq})}^{2-}$$

5 $$2{\mathrm{H}}_{2}\mathrm{O}+2{\mathrm{e}}^{-}\to 2{\mathrm{OH}}^{-}+{\mathrm{H}}_{2(\mathrm{g})}$$

The carbonic acid required in the cathode reaction comes from the dissolution of CO_2_ in the NACE solution, so that the dissolution and mass transfer process of CO_2_ in the cathode reaction play a vital role in the reaction process:

6 $${\mathrm{CO}}_{2(\mathrm{g})}\leftrightarrow {\mathrm{CO}}_{2}$$

7 $${\mathrm{CO}}_{2}+{\mathrm{H}}_{2}\mathrm{O}\leftrightarrow {\mathrm{H}}_{2}{\mathrm{CO}}_{3}$$

The anode reaction mainly include^19^:

8 $$\mathrm{Fe}+{\mathrm{H}}_{2}\mathrm{O}\to {\mathrm{FeOH}}_{\mathrm{ads}}+{\mathrm{H}}^{+}$$

9 $${\mathrm{FeOH}}_{\mathrm{ads}}\to {\mathrm{FeOH}}^{+}+\mathrm{e}$$

10 $${\mathrm{FeOH}}^{+}+{\mathrm{H}}^{+}\to {\mathrm{H}}_{2}\mathrm{O}+{\mathrm{Fe}}^{2+}$$

Fe^2+^ generated by the dissolution of the metal matrix, and the concentration gradually increases near the wall with the corrosion progresses. At the same time, the consumption of H^+^ in the anodic reaction leads to the gradual increase of the concentration of ${\text{CO}}_{{3({\text{aq}})}}^{2 - }$(or ${\text{HCO}}_{{3({\text{aq}})}}^{ - }$), and some corrosion products will deposit when the concentration reaches the limit concentration of FeCO_3_^20^.

11 $${\mathrm{Fe}}^{2+}+{\mathrm{CO}}_{3}^{2-}\to {\mathrm{FeCO}}_{3}$$

12 $$\mathrm{Fe}+{\mathrm{HCO}}_{3}^{-}\to {\mathrm{FeCO}}_{3}+\mathrm{H}$$

The control process of corrosion medium on the corrosion process mainly includes the dissolution rate of CO_2_, the mass transfer and ionization rate of H_2_CO_3_, and the mass transfer rate of H^+^. For the corrosion in static solution, the dissolution process of CO_2_ gas is slow because the interface between CO_2_ gas and solution remains relatively static. Therefore, the dissolution rate of CO_2_ and the mass transfer and ionization rate of H_2_CO_3_ are the main control steps of the corrosion process.

### Corrosion reaction intermediate product

EIS showed that the minimum charge transfer resistance of each group appeared at the beginning of corrosion reaction, and the charge transfer resistance under different liquid film depths all increasing with the corrosion time. Which is proved that the concentration of ions in the solution is not affected by the liquid film thickness at the initial stage of the corrosion, and the ions near the wall contact with the metal matrix and react through diffusion and mass transfer. The ion concentration of the corrosion medium near the wall decreases and the ion concentration of the corrosion product increases with the corrosion progress. The ion movement in the static solution only depends on the diffusion mass transfer, which resulting in a gradual increase of the charge transfer resistance and the decrease of the corrosion rate. Under this condition, the corrosion rate is affected by many factors, including the diffusion and mass transfer rate of corrosive medium ions in the main solution to the solid–liquid interface, the rate of CO_2_ gas dissolution and the ionization of H_2_CO_3_, and the influence of the rising concentration of corrosion product ions in the solution on the dissolution rate of CO_2_.

The inductive loop is usually related to the adsorption and desorption of corrosion intermediates or the stripping of corrosion products on the test surface, but corrosion products are difficult to be stripped in static condition. The corrosion mechanism show there is intermediate product FeOH in the CO_2_ corrosion of X80 steel, and the process of adsorption and desorption will lead to the emergence of inductive loop. In the later stage of corrosion, the characteristics of inductive loop gradually disappear, which is mainly due to two reasons: on the one hand, H^+^ is continuously consumed and the concentration of H^+^ decreases with the corrosion progress, which leads to the increase of solution pH and weakens the phenomenon of intermediate product adsorption or pitting nucleation at low pH (pH ≤ 3)^21,22^; on the other hand, the concentration of corrosion intermediates increased with the corrosion progress, which increased the coverage of the intermediates on the test surface. Therefore, with the increase of the liquid film thickness, the total amount of initial corrosion medium ions in the solution with the same concentration also increases. Meanwhile, the pH and the concentration of corrosion medium ions slow down with the decline of corrosion reaction, which leads to the extension the time of inductive loop.

### Local corrosion

Under the static liquid film, the liquid film thickness inside ADP is deeper than that outside plane of ADP, so the distance from the CO_2_ solution gas–liquid interface above the liquid film is farther. At the initial stage of the corrosion, the ion species and concentration at everywhere of the solid–liquid interface are consistent, which promotes the same corrosion reaction inside and outside of the ADP. Therefore, the SEM shows that there are corrosion products inside and outside ADP. However, the CO_2_ in ADP is consumed quickly by the corrosion reaction, and it is difficult for dissolved CO_2_ to quickly reach the ADP through diffusion mass transfer because the relatively long mass transfer distance from the gas–liquid interface. Therefore, the cathodic reaction inside ADP is terminated after CO_2_ consumption, and then the anodic reaction become to the main reaction^23^. The relatively close mass transfer distance outside ADP makes part of the dissolved CO_2_ reach the plane area through diffusion mass transfer, so the cathodic reaction in the corrosion reaction mainly occurs in this area. When the anodic reaction dominate inside ADP, the concentration of metal cations gradually increases with the progress of corrosion. Also, Cl^-^ begin to gather in ADP because the charge balance causes, and the hydrolysis reaction of Fe increases the concentration of H^+^ and enhances the acidity inside ADP^24^. These comprehensive factors lead to the process of "depletion of CO2 in the pit → termination of cathodic reaction → anodic reaction → increase of Cl—concentration in the pit → hydrolysis of metal ions → acidification in the pit → accelerated dissolution of matrix" inside ADP, which is called "autocatalytic acidification" in the local corrosion pit. Therefore, the corrosion in the ADP is more serious than the plane out of ADP^25^.

However, the 3D morphology shows that there is no crevice corrosion or pitting corrosion in test surface. The reason is that the outside ADP mainly acts as the cathode in the corrosion, and there is only partial corrosion at the initial stage of the corrosion, so the metal matrix is basically in a uniform corrosion state. Although the inside ADP is act as anode in the corrosion, but without interference in the laboratory the corrosion enhancement effect tends to be uniform. The corrosion inside ADP can be considered as a local corrosion process of overall enhancement compared with the whole test surface. In fact, there are various interference factors such as other corrosive gases, solid particles, exfoliated corrosion products and other substances will destroy the corrosion balance in static solution, thus inducing smaller scale pitting corrosion, crevice corrosion and so on.

## Conclusions

The main conclusions are as follows:

1. Under the different static liquid film thickness (≥ 1 mm) studied in this paper, the corrosion mechanism of X80 pipeline steel is basically the same, and the liquid film depths only affect the reaction time of each step of the corrosion. The adsorption, desorption and coverage of corrosion intermediates (FeOH) will cause the inductive loop. With the corrosion progress, the thicker liquid film lead to the change of ion concentration more slowly, and the increase of pH is slow down also, thus prolonging the appearance time of inductive loop.
2. The CO_2_ corrosion progress of X80 pipeline steel under the static liquid film can be divided into two stages: in the early stage, the corrosion is uniform because the ion concentration is the same at test surface; in the later stage, due to the influence of CO_2_ dissolution and mass transfer distance, the cathode reaction is mainly outside ADP, and the anode reaction is mainly inside ADP. The hydrolysis reaction of Fe leads to the increase of H^+^ concentration, the decrease of pH in the ADP, and the accumulation of anions. Which will cause the corrosion autocatalytic acidification occurred in the ADP, and accelerated the corrosion process in the ADP.
3. In the laboratory, the corrosion acceleration in the ADP is uniform, and there is no smaller scale point corrosion or crevice corrosion. Therefore, the corrosion enhancement process in the defect pit is considered as a local corrosion process of overall enhancement.

## Data Availability

The raw/processed data required to reproduce these findings cannot be shared at this time as the data also forms part of an ongoing study.
